# We Can Boost IQ: Revisiting Kvashchev’s Experiment

**DOI:** 10.3390/jintelligence8040041

**Published:** 2020-11-26

**Authors:** Lazar Stankov, Jihyun Lee

**Affiliations:** 1 School of Psychology, The University of Sydney, Sydney, NSW 2006, Australia; 2 School of Education, The University of NSW, Sydney, NSW 2096, Australia; jihyun.lee@unsw.edu.au

**Keywords:** intelligence, training, creative problem-solving

## Abstract

This paper examined the effects of training in creative problem-solving on intelligence. We revisited Stankov’s report on the outcomes of an experiment carried out by R. Kvashchev in former Yugoslavia that reported an IQ increase of seven points, on average, across 28 tests of intelligence. We argue that previous analyses were based on a conservative analytic approach and failed to take into account the reductions in the IQ test variances at the end of the three-years’ training. When standard deviations of the initial test and 2nd retest were pooled in the calculation of the effect sizes, the experimental group’s performance was 10 IQ points higher on average than that of the control group. Further, with the properly defined measures of fluid and crystallized intelligence, the experimental group showed a 15 IQ points higher increase than the control group. We concluded that prolonged intensive training in creative problem-solving can lead to substantial and positive effects on intelligence during late adolescence (ages 18–19).

## 1. Introduction

For several decades, hereditarians have shaped our beliefs about the modifiability of intelligence. Jensen (1969) argued that the heritability estimate of intelligence is about 80% and that the role of the environment or schooling has to be small and perhaps negligible. The argument supported the claim that the effects of compensatory early education on preschool children tend to be low. This led not only to an increased focus on IQ differences across racial/ethnic groups, but also to the acceptance of the view that training of cognitive performance is likely to be ineffective. Training effects are, at best, limited to the practiced tasks (Jensen 1969), while far transfer—i.e., passing of knowledge and skills from the taught context to the distantly related tasks or the processes captured by a broader *g* factor—is unlikely to be achieved. 

Until recently, Jensen’s views about heritability and training effects have been influential within the intelligence research community (see Eysenck 1971; Haier 2014). Nowadays, it is generally accepted that the heritability of intelligence increases from about 20% in infancy to perhaps 80% in later adulthood (Plomin et al. 2014; Plomin and Deary 2015). This leaves plenty of room for environmental effects, especially within the school-aged populations.

## 2. Theoretical Background

### 2.1. The Effect of Cognitive Training on Intelligence

There are continuing debates about the effects of training on cognitive abilities. In 2014, two groups of scientists put forward opposing views (see Simons et al. 2016). One group claimed that there is no compelling scientific evidence that cognitive training can reduce or reverse cognitive decline. Of particular interest was the claim that training on working memory can improve fluid intelligence, which attracted a lot of criticism (see Novick et al. 2020). The other group was stating that cognitive training procedures can significantly improve cognitive function.

Harvey et al. (2018) belongs to the positive camp, arguing that computerized cognitive training is effective for far transfer, i.e., it can improve cognitive performance on untrained skills in both healthy older people and people suffering from schizophrenia. This is reminiscent of the findings from previous work that focused on practicing performance with competing/dual cognitive tasks (see Fogarty and Stankov 1982, 1988). It was found that doing two intelligence tests simultaneously—e.g., one presented through the earphones and another on the computer screen—for eight practice sessions led to both overall improvement in all components of the competing tasks and also to an increase in the common variance captured by tests of fluid intelligence (Stankov 1991). Therefore, depending on the choice of the cognitive tasks for practice and the training protocol, the practice may lead to transfer effects beyond the closely related tasks.

On the other hand, Simons et al. (2016) were negative and agreed with Jensen about the poor effectiveness of the far transfer. They concluded that there is extensive evidence that brain-training interventions improve performance on the trained/practiced tasks, but less evidence that such interventions improve performance on closely related tasks, and further, “…little evidence that training enhances performance on distantly related tasks or that training improves everyday cognitive performance” (p. 103). Overall, the conclusion was rather pessimistic. They also reported that many of the published intervention studies had major shortcomings, including small sample size, short periods of training, lack of random assignment and pre-test baseline, reliance on a single measure of intelligence, and the absence of a control group. Stankov (1986), however, pointed out that most of the shortcomings mentioned above do not apply to the experimental conditions of Kvashchev’s (1980) study because it had a relatively large sample size (total N close to 300), lasted over three years, relied on 28 measures of intelligence, and it was based on random assignment of school classes to the control and treatment groups. Given the positive outcomes of Kvashchev’s experiment, it was suggested that the carefully designed training can be effective if it is long-lasting and intensive.

Recent studies have also challenged Jensen’s (1969, p. 2) claim about the failure of compensatory education in boosting intelligence. For example, a meta-analysis reported an increase in intelligence test scores between one and five (3.4 IQ points on average) for one additional year of schooling (Ritchie and Tucker-Drob 2018). This finding was replicated in a large-scale study by Hegelund et al. (2020) in Denmark, who found an average increase in intelligence test scores of 4.3 IQ points per year of education in young adulthood and 1.3 IQ points in adults in mid-life. They also reported that when taking the intelligence test performance at age 12 into account, the individuals with low intelligence in childhood derived the largest benefit, concluding that education constitutes a promising method for raising intelligence, especially among the individuals from a disadvantaged background. Although these studies did not examine specific aspects of the compensatory intervention, the evidence suggests that more years of formal schooling can improve the cognitive functioning of children and young adults.

The strong hereditarian position has also been challenged in studies that investigate the role of socioeconomic status (SES) in the development of intelligence. For example, Selita and Kovas (2019) argued that social inequality and social policies can have a profound effect on the heritability of educational attainment in the general population. They showed that heritability is higher among the people living in neighbourhoods with greater equality levels, implying that social inequality conditions stifle the expression of educationally relevant genetic propensities. This appears to agree with the findings of Weiss and Saklofske (2020), who focused on environmental rather than hereditary influences on group differences in intelligence. They reviewed the findings with several standardized IQ tests and suggested that the pronounced environmental effects on IQ, in addition to those captured by the SES, can be identified within the socially disadvantaged samples of African Americans. 

A meta-analysis reported by Ma (2009)^1^ included 111 empirical studies on creativity to examine the effects of a host of psychological (e.g., personality, motivation, leadership, self-efficacy, and emotional stability) and environmental (e.g., teacher encouragement, peer competition, satisfaction with class climate, and pedagogical approaches) variables on four types of creative performance: nonverbal creativity, verbal creativity, problem-solving creativity, and emotional creativity. Two findings are particularly pertinent to the present study. First, the mean effect size was far stronger for problem-solving creativity (Cohen’s d = 0.86) than it was for verbal creativity (0.79), nonverbal creativity (0.45), and emotional creativity (0.34). Second, when the effect sizes were calculated for five different components of creative problem-solving process (defining a problem, retrieving relevant knowledge, generating solutions, evaluating, and selecting solutions), the strongest effect (0.93) was found for the component of defining a problem, which included the processes of restating the problem in as many different ways as possible before beginning to solve it. Overall, Ma (2009) reported larger than medium weighted average effect size of 0.72 for these five components.

### 2.2. Kvashchev’s Experiment

Jensen’s views were challenged by Stankov (1986). He reported findings from one of a series of intervention studies carried out by a Yugoslavian educational psychologist R. Kvashchev (1980). The intervention was conducted in the mid-1970s in two high schools in a small town in northern Serbia. One school was treated as control (N = 147, with five classes selected randomly, representing about 50% of the student population of the school), while the other school was designated as experimental (N = 149, with five classes, also selected randomly to represent about 50% of the school). The experiment started with the first-year high school students (on average 15 years old) following eight years of primary schooling. Students at the experimental school were given special classes in creative problem-solving. Such classes were offered at least once a week and teachers were trained by Kvashchev himself to develop creative thinking exercises for their courses in specific school subject areas (e.g., mathematics, science, Serbian language). 

Stankov (1986, pp. 212–19) provided detailed information about the experimental procedure, training exercises, and descriptions of the 28 tests of intelligence that the students were tested on. Thus, only a summary version is presented herein. All exercises used in Kvashchev’s experiment were referred to as training in “creative problem solving”. The principles employed in the construction of the training exercises included the requirements that: (a) exercises should call for a combination of elements that are remote rather than close in terms of their associational value; (b) exercises should call for a radical reorganization and reformulation of the problem situation to achieve a satisfactory solution; and (c) exercises should call for both convergent and divergent thinking operations, especially the latter.

Kvashchev collected the exercises involving creative thinking and problem-solving processes that were available in textbooks or journal articles over the previous decades and presented them to the experimental group, either in their original form or, if necessary, translated or developed in collaboration with the teachers to conform to the syllabus of a particular school subject. To illustrate, consider the following problem:

“I was captured by a band of outlaws—said a famous explorer—and their leader had my hands and legs tied up so that 1could not move. They did not gag me up though, and I was able to use my mouth freely. The leader of the gang hung a piece of bread exactly five centimetres away from my mouth. He then laughed and said: "If you manage to eat this piece of bread, I’II set you free. He knew that I could get no help. Also, in order to ensure that I cannot roll over or move closer to bread, they tied me to a tree. Nevertheless, I managed to free myself. How?”

Students in the experimental group were asked to list as many ways as they could think of to solve the problem. The most salient features of this type of exercise were students’ active participation and their attention to the principles deemed important for creative thinking, that is, production of as many imaginative solutions as one can come up with. The acceptable solutions for the example above include blowing at the bread to create a pendulum or solutions that assume some particularly favourable conditions, such as the wind blowing and moving either the rope or the tree (or both) to which the explorer is attached.

Students in the experimental school were given such creative problem-solving exercises at least once-a-week over the next three years. Students in the control group, on the other hand, attended the school in a typical way over the same period. The students in both (experimental and control group) schools completed a battery of 28 intelligence tests, such as matrices, verbal analogies, and number series, when they enrolled in the high school (see Appendix A for the complete list of 28 tests). All cognitive tests were subtests from verbal and nonverbal IQ test batteries and are known to have acceptable reliabilities, ranging between 0.65 and high 0.80 (Kvashchev 1980). Six nonverbal tests were parts of Cattell’s culture fair battery and five were part of the Bujas’ test of intelligence. The well-known nonverbal Dominoes D-48 test was also employed. Two verbal tests consisting of several subtests were presented in Serbo-Croatian. One verbal test was developed by Zoran Bujas, a student of an eminent French psychologist Henry Pieron, and the other test was developed by Borislav Stevanovic, a former student of Charles Spearman.

The first round of test administration was referred to as the “Initial Test”. All participants were given the same set of 28 intelligence tests again at the end of the experiment, when students were in their 3rd year of high school (on average aged 18). This was referred to as the “Final Test”. Further, at the beginning of the next and final high school year, students (on average aged 18.3) in both schools took the same intelligence tests again (“1st Retest”). No training in creative problem-solving was given in the last year of high school. However, at the very end of the high school (on average, aged 19), they were tested again with the same battery (“2nd Retest”).

It is important to keep in mind that creative problem-solving exercises were not designed to practice or train cognitive processes measured by the set of 28 intelligence tests. As illustrated above, the principles that guided the construction of these exercises called for a combination of elements that were remote rather than close in terms of their associational values and required a radical reorganization and reformulation of the problem situation to achieve a satisfactory solution. In other words, the expected answers were heavily dependent on divergent thinking processes. Thus, it is reasonable to assume that the changes in the performance on 28 tests of intelligence, which in fact relied largely on convergent thinking processes, can be attributed to the presence of far transfer.

**Figure 1:**
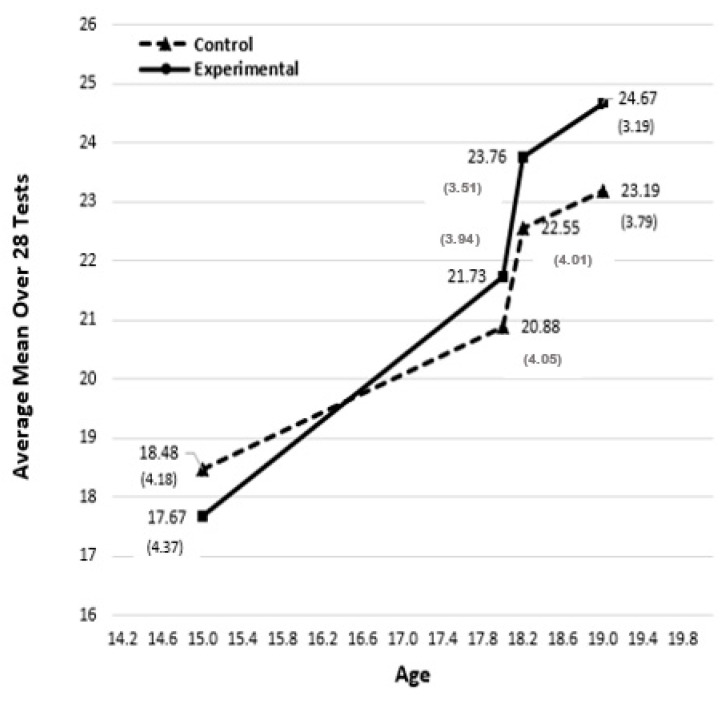
Arithmetic means over 28 cognitive tests from Stankov’s (1986, Table 1) at the Initial (age 15), Final (age 18), 1st Retest (age 18.3), and 2nd Retest (age 19) occasions. Standard deviations are in brackets.

Stankov (1986, Table 1) presented the means and standard deviations for each of the 28 tests on four occasions of testing (Initial, Final, 1st Retest, and 2nd Retest). In Appendix A of this paper, the first (Initial) and last (2nd Retest) of the same dataset have been reproduced. We calculated the averages (means) for both experimental and control groups across the 28 tests in the four occasions of testing in Figure 1 and the last row in Table A1 in the Appendix A. Most test results displayed similar patterns of the means and standard deviations over the four occasions, and thus only the averages over the 28 tests are plotted in Figure 1 (see also Stankov 1991). Averages (means) over all the 28 tests can be interpreted as a global performance measure of intelligence because the individual subtests capture different aspects of general cognitive ability. Most tests can be classified either as measures of fluid (Gf) or crystallized (Gc) intelligence (see Table A1 in the Appendix A).

In the initial stage of the experiment at age 15, the control group had a higher average score than the experimental group. There was a crossover in the performance at the end of the training and the experimental group became superior at age 18 (Final). As can be seen in Figure 1, this superiority of the experimental group became even stronger in the retest stages at ages 18.3 (1st Retest) and 19 (2nd Retest). Analysis of covariance (ANCOVA) with the individuals’ scores at the initial stage as the covariate led to statistically significant F-values, with the experimental group showing better performance on 26 out of 28 tests in the 2nd Retest session (i.e., the last testing; also reported in Stankov 1986, Table 2). Thus, the overall conclusion was that the experiment produced statistically significant and positive results.

Specifically, the improvement of the experimental group at age 18 was, on average, 5.66 IQ points higher than the control group. A year later at age 19 (2nd Retest), the improvement of the experimental group was, on average, 7 to 8 IQ points higher than the control group. In terms of Cohen’s *d* effect size criteria, the change represents a ‘medium’ (0.50) effect size (Cohen 1988). This led to the cautious conclusion that through training in creative problem-solving it is “… possible to achieve small improvement in performance” (Stankov 1986, p. 209). Given the nature of the experiment and the wide range of the test batteries, it was believed that far transfer was demonstrated one year after the end of the treatment (at age 19). However, the overall effect was not seen as sufficiently strong, which led to the questioning tone in the title of Stankov’s (1986) paper, “*Can we boost intelligence?*”

## 3. Methodology and Results

### New Insights and Proposed Reanalysis

While the findings of Stankov (1986) were generally accepted by the intelligence research community, the questioning tone of the title led to the contribution being seen as inconclusive. However, there were three aspects of the analyses that appear to have been too conservative. First, two ANCOVA analyses were carried out on each of the 28 tests in the battery. One was based on individuals and the other was based on class as the unit of analysis. The latter approach had a small number of degrees of freedom and, consequently, it did not identify strong effects on several tests that were statistically significant in the analyses based on individual-level data. Problems related to low degrees of freedom using the class as the unit of analysis were not sufficiently acknowledged in Stankov (1986). Second, the interpretation focused on the student performance data at the end of training (i.e., “Final”) and less so on the 2nd Retest, although the latter showed the longer-lasting and stronger far transfer effects (see Figure 1).

Third, and most importantly for our purposes here, the standard deviations from the Initial testing were employed in the calculation of the effect sizes and their IQ equivalent scores. The use of pre-test information about dispersions was justified by the fact that students’ performance before the start of the training can be seen as representative of the population. In Kvashchev’s (1980) experiment, however, the students’ performance in both experimental and control groups was more heterogeneous at the beginning of training and the standard deviations were significantly larger at the beginning of training than at the end of the experiment. 

In the discussion section of his book, after rejecting the participants’ attrition as a likely cause for his finding, Kvashchev (1980) made note of the two aspects of the reduced post-test variance. First, both experimental and control groups benefitted from the four years of high-school experience, but the training effects were stronger in the experimental group, especially on participants with low cognitive abilities at the start of the experiment. Second, the high achieving students might have approached a ceiling level on some intelligence tests he had employed which, again, was more pronounced in the experimental group. 

Lower heterogeneity of the experimental group at the end of treatment is important for our re-analysis of the effect sizes. The top part of Table 1 shows calculations that are analogous to those used in Stankov (1986, p. 228) to arrive at the 7 to 8 IQ points differences between the experimental and control groups on the Initial and 2nd Retest, as mentioned above. In other words, standard deviations (4.37 and 4.18) from the Initial testing session were used to arrive at the 7.13 difference in IQ points, leading to what we now believe to be a conservative result. Using the values of the Initial test alone in the calculation of the effect size had disregarded smaller variances obtained after four years of schooling by both control and experimental groups, particularly in the latter. For comparison, the same statistics were calculated using the 2nd Retest standard deviations (3.19 and 3.79), instead of the values at the Initial session. These are presented in the middle section of Table 1 and the difference in the effect sizes expressed in IQ units is now 14.28, about twice as large as what was reported in Stankov (1986).

The use of Cohen’s *d* for the calculation of the effect size has become common because of the proliferation of meta-analyses and a need to have comparable statistics across different empirical studies. Consequently, the practice of using pre-test standard deviations alone for the calculation of the effect sizes, in the within-groups design, is much less frequently seen today. Cohen himself recommended a formula that was analogous to the t-test calculation in the within-subjects design. Thus, the standard deviation that was used in the calculation of what is labelled as Cohen’s *d_z_* was based on the pooled variance—i.e., the sum of the pre-test and post-test variances, as well as on the correlation between the two occasions of testing. In fact, the effect size can be calculated directly from the repeated measures t-test (see Lakens 2013, p. 4). However, since meta-analyses may consider both between- and within-subjects designs Cohen’s *d_z_* is also infrequently used today.

Cumming (2012) suggested that the calculation of the effect size in the within-subjects designs can be based on the average of the standard deviations from both occasions—i.e., neglecting correlations between the occasions of testing. He referred to this value as Cohen’s *d*_av_ (see Lakens 2013, p. 5). This procedure and results with Kvashchev’s battery of 28 tests are illustrated in the bottom row of Table 1 (see also the “AVERAGE” row in Table A1 in the Appendix A). The experimental group showed about 10 IQ points stronger effect size (Cohen’s *d_av_* = 0.67) than the control group. In intelligence research where an IQ score of 15 points is equal to one standard deviation, a 10-point difference cannot be classified as small improvement.

When considering the results for each of the 28 tests (see the Appendix A), the experimental group did show superior performance compared to the control group on all but one test, specifically, the arithmetic test where the difference was virtually zero (−0.27 in IQ points). The IQ difference between the experimental and control groups was the largest (19.49 IQ points) for the word classification test, while six tests showed a difference of 15 IQ points or higher (see the last column in the Appendix A). We believe that in the within-subjects studies with pronounced differences between the pre-test and post-test variances, the effect sizes expressed in terms of Cohen’s *d_av_* may be more appropriate than those based on the pre-test variances only.

It is necessary to point out that even though the tests employed in Kvashchev’s studies were part of intelligence test batteries, the scores that were used in the analyses in this paper are raw scores and not the normed IQ scores. Instead, the difference between the experimental and control groups are analogous to Cohen’s *d_av_* values that were rescaled to correspond to the typical IQ metric.

## 4. Changes in Fluid (Gf) and Crystallized (Gc) Intelligence

As can be seen in the last column of the Appendix A, some tests showed stronger effect sizes than the others. It may be reasonable to ask whether the difference across all 28 tests can be attributed to fluid (Gf) or crystallized (Gc) intelligence, and, if so, whether the training was more effective for Gf or Gc. Two papers by Stankov and Chen (1988a, 1988b) used the data from Kvashchev’s experiment and employed structural equation modelling (SEM) to assess factorial invariance. Due to the limited computational power in the 1980s, the studies were based on different selections of tests (11 tests in [1988a] and eight tests in [1988b]) from the list of 28. The choice of tests for each SEM study was based on the putative cognitive processes captured by the test. For example, in Stankov and Chen (1988a), six tests from the Cattell’s culture fair battery (see tests 23–28 in the Appendix A, Table A1) were chosen as measures of Gf, while proverbs, verbal analogies, word classification, essential features, and disarranged sentences were chosen as potential measures of Gc. Stankov and Chen (1988b) was based on a different selection of tests and there was no overlap between the two studies. Since a priori classification of the remaining nine tests was less clear-cut in terms of Gf–Gc structure, they were not used in the SEM analyses. In both studies, separate Gf and Gc factors were fitted and it was shown that the factor structure was invariant across the experimental and control groups.

Using the information on factor score means in Stankov and Chen (1988a, 1988b) and the procedures analogous to those presented in the bottom part of Table 1, the training effects can be summarized in the following way. The calculations based on Stankov and Chen’s (1988a) report indicate that the training led to an increase of about 10 IQ points on the Gc factor and about 27 IQ points on the Gf factor. On the other hand, the calculations based on Stankov and Chen’s (1988b) analyses show that the training led to the increase of about 21 IQ points on the Gc factor and only five IQ points on the Gf factor. Overall, across the two studies, the change was about equal on the two factors (10 + 21)/2 = 15.5 IQ points on Gc, and (27 + 5)/2 = 16 IQ points on Gf. In both cases, the improvement was above 15 IQ points^2^.

Nevertheless, as can be seen from the last column in the Appendix A, the selection of tests may make quite a difference to the results. A closer look at the tests themselves may provide useful clues. For example, as mentioned above there was a pronounced difference in the Gf factor in two Stankov and Chen (1988a, 1988b) studies (27 IQ points in the 1986a study and five points in the 1986b study). The five Gf tests in the Stankov and Chen’s (1988a) study were all from the Cattell’s culture fair battery, a well-known test of fluid intelligence. On the other hand, only one of the Gf tests in Stankov and Chen’s (1988b) study (Dominoes-48 test) was an established Gf marker. The other three—perceptual reasoning test, multiple solutions tests, and pictorial poly-profile test—appear to contain pronounced visual perception content in addition to the Gf component. It is plausible that the perceptual processing component may have led to a less pronounced Gf effect in Stankov and Chen (1988b).

## 5. Conclusions and Discussion

It can be concluded that Stankov’s (1986) account of the effects of Kvashchev’s training in creative problem-solving on the general factor of intelligence may have been too conservative. The reanalyses presented in this paper indicate that the experimental group gained at least 10 IQ points more than the control group at the end of the four years. Further, on some cognitive tests (see Appendix A) and on the properly defined measures of fluid and crystallized intelligence (Stankov and Chen 1988a, 1988b), the advantage of the experimental group was more than 15 IQ points. The effects can be classified as ‘upper medium’ or ‘large’, following Cohen’s effect size guideline (Cohen 1988). There were two additional training studies—one devoted to the development of creativity and the other on critical thinking—carried out by Kvashchev over a 27-year period that produced similar outcomes, but at the time of his death in 1983 the actual data were available only for the study reported herein. 

Several implications can be drawn in the context of the current views about intelligence and the nature of cognitive training and schooling. First, an important observation was the reduction of the test variances that took place under the influence of training. This may be particularly pronounced in long-lasting interventions during the school years of childhood and adolescence. Kvashchev (1980) suggested that the reduced variances may be partly due to the increase in performance (especially among the initially lower-scoring participants) and partly due to reaching the ceiling levels of the tests employed (especially by the highly able participants). This reduction in dispersion, perhaps due to the ceiling effect, was evident at all successive retesting sessions, but especially at the later stages of retesting (see standard deviations in Figure 1). An important lesson from this work points to the need to employ tests of a sufficient range of difficulty levels in similar studies in the future. A realistic assessment of the changes in the cognitive abilities of young students would also need to consider the role of maturation in the reduced variances in both experimental and control groups. 

Aside from reduced variances, there is clear evidence of an increase in the mean levels of performance across the occasions of testing. Further, that increase was statistically significant in both experimental and control groups (see Stankov 1986). Compared to the control group, the effect in the experimental group was larger—a difference of 10 to 15 IQ points—indicating that training in creative problem-solving can improve performance on tests of intelligence. Of particular interest to educationists may be the observation that there was a larger increase in the experimental group’s performance a year after the end of the training. Longitudinal studies will be needed to examine longer-lasting effects of training.

Second, Sauce and Matzel’s (2018) views about the gene–environment interplay may be relevant to this discussion. They postulated that the malleability of intelligence exists and conclude that “… one can say that IQ has high heritability and a high malleability”. Their evidence was mostly observational and included IQ gains consequent to adoption/immigration, changes in heritability across the lifespan and socio-economic status, the Flynn effect, the slowdown of age-related cognitive decline, and IQ gains via early compensatory education. Kvashchev’s work on the effects of training in creative problem-solving provides experimental evidence for the important role of the environment.

Third, the present study adds valuable information to an ongoing debate within medicine, information technology, and education, as well as the theoretical cognitive psychology, about the effects of training (see Simons et al. 2016; Harvey et al. 2018). Kvashchev’s work shows that with the prolonged and intensive training in the school environment, far transfer is possible in the cognitive domains that have not been deliberately included in the training protocol.

Critics of Kvashchev’s work may argue that the experiment was not carried out in a laboratory setting and that Kvashchev’s school-based experiment is inferior to more conventional laboratory interventions because the trained processes were not explicitly defined and thus the effects cannot be unambiguously attributed to specific treatments. In the natural environment, it was not possible to control all or many potentially confounding variables, and different aspects of participant schools might have also played a role in addition to that of creative thinking exercises. The school environment factor is an important and relevant aspect of the debate since laboratory-based training over several weeks (despite carefully crafted exercises) has produced controversial or mixed outcomes. The Kvashchev’s experiment results, based on the comparison to the control group that was similar to the experimental group, coupled with the prolonged training over three years conducted in the natural environment, the presences of the pre-test measures, and the employment of a variety of 28 IQ tests, cannot be easily dismissed. We may further claim that the experiments conducted in the actual classroom, on a weekly basis, involving collaboration between the school teachers and the researcher, in fact, strengthen the external validity argument.

Contemporary studies involving cognitive performance training often do not contain many subtests of WAIS or WISC, let alone as many as 28 tests employed here. Such studies often rely on a single test, like the Raven’s Progressive Matrices. The evidence collected from many tests is certainly a better approximation of *g* than a single or smaller number of tests (Gignac 2015). With a variety of cognitive tests, one can expect some variation in the training effects. Stankov and Chen’s (1988a, 1988b) studies indicate that the broad Gf and Gc factors happen to be about equally affected by Kvashchev’s training. However, the differences in the effects of training on Gf factors suggest that visual perceptual processes may be pronounced in one set of Gf tests but less so in the other. Thus, it is useful to examine closely varying degrees of training outcomes on a range of different cognitive tests.

Some contemporary theorists also do not attach particular importance to *g*. For example, some adherents of the Cattell–Horn–Carroll (CHC) theory have pointed out that the percentage of the variance accounted for by the first principal component is small and can be neglected in favour of the broad factors, such as Gf and Gc (Stankov 2019a). Another group of theorists view *g* as a formative construct rather than a reflective “source trait”. For example, in Kovacs and Conway’s (2019) process overlap theory (POT), intelligence was interpreted in a way similar to socio-economic status, i.e., different indices (tests) contribute to its formation but there is no psychological entity underlying it. Although it is not clear how the increase in cognitive performance following the training would be interpreted under this formulation, the importance of IQ is also diminished by the POT (Stankov 2019b).

In conclusion, we argue that cognitive abilities captured by the tests of intelligence may not be fixed entities, since prolonged and intensive training in creative problem-solving within typical school environments can lead to sizable and positive gains in the overall cognitive function in late adolescence (ages 18–19). It may be argued that future work needs to focus on theoretical issues, such as the identification of elementary cognitive processes that facilitate gains. An alternative direction may involve practical topics, such as validation of the effects of training in creative problem-solving on real-life situations beyond IQ tests. Finally, training based on computerized games, machine learning, and artificial intelligence may benefit from the use of principles incorporated in creative problem-solving exercises.

## Figures and Tables

**Table 1:** Differences between the Initial (age 15) and 2nd Retest (age 19) over 28 cognitive tests.

Analytic Approach	Group	Difference between the Means at Initial & 2nd Retest	Standard Deviation	Training Effect Size in Cohen’s *d_av_*	Group Difference in Terms of Cohen’s *d_av_*	Training Effect Size × 15 in IQ Units	Group Difference in IQ Units
Stankov (1986)	Experim.	7.00 (24.67–17.67)	4.37	1.60	0.47	24.00	7.13
	Control	4.71 (23.19–18.48)	4.18	1.13		16.90	
Recalculation using 2nd Retest standard deviations	Experim.	7.00	3.19	2.19	0.95	32.92	14.28
	Control	4.71	3.79	1.24		18.64	
Recalculation using the average of pretest–post-test standard deviations (Cumming 2012; Lakens 2013)	Experim.	7.00	3.78 (4.37 + 3.19)/2	1.85	0.67	27.78	10.08
	Control	4.71	3.99 (4.18 + 3.79)/2	1.18		17.70	

Note. “Training effect sizes” are equivalent to different versions of Cohen’s *d_av_* (see Lakens 2013). They were calculated as ratios between “mean difference between 2nd Retest and Initial” (column 1) and corresponding “standard deviation” (column 2).

## Footnotes

1 We are grateful to an anonymous reviewer who alerted us to the Ma’s (2009) meta-analysis.

2 We also calculated effect sizes using standard deviations from the initial testing session, reported in the Stankov and Chen (1988a, 1988b) studies, i.e., in the way it was done on the first row of Table 1. This showed the overall 10 IQ points superiority of the experimental group on the Gc factor and 16 points superiority on the Gf factor.

## Appendix A

**Table A1:** Means and Standard Deviations from Stankov’s (1986) Table 1 for the control and experimental groups on the Initial and 2nd Retest Occasions and effect sizes in IQ Units.

IQ Tests	Control Group					Experimental Group					Difference (Experimental–Control) in IQ Units
	Initial (Age 15)		2nd Retest (Age 19)		Effect Size in IQ Units	Initial (Age 15)		2nd Retest (Age 19)		Effect Size in IQ Units	
	Mean	SD	Mean	SD		Mean	SD	Mean	SD		
1. Essential Features (Gc)	30.76	5.45	37.49	4.21	20.90	28.41	6.72	39.42	2.83	34.59	13.69
2. Word Classification (Gc)	10.84	2.52	12.84	3.51	9.95	10.05	2.76	14.29	1.56	29.44	19.49
3. Number Series	10.61	2.37	12.46	2.14	12.31	9.87	2.44	13.77	1.81	27.53	15.22
4. Consequences (Gc)	34.90	4.92	38.33	3.29	12.53	31.91	6.41	39.21	3.38	22.37	9.84
5. Relations (Gc)	12.56	2.63	14.87	1.66	16.15	10.59	3.04	15.15	1.21	32.19	16.03
6. Word Meanings	28.78	6.83	37.32	5.02	21.62	26.63	7.48	39.20	4.25	32.15	10.53
7. Unbalanced Structures (Gc)	10.28	2.53	13.19	2.08	18.94	8.85	2.62	13.66	1.73	33.17	14.24
8. Numeric	14.08	2.01	14.88	1.11	7.69	13.60	2.07	15.12	1.11	14.34	6.65
9. Pictorial Poly-profile	23.00	6.67	33.01	6.33	23.10	20.53	6.00	35.33	5.23	39.54	16.44
10. Meaningful Memory	12.96	4.55	21.72	8.59	20.00	13.81	5.53	25.91	8.63	25.64	5.64
11. Word Classification “S”	14.29	2.79	18.59	2.97	22.40	15.46	2.79	20.82	2.26	31.84	9.45
12. Proverbs (Gc)	9.50	3.93	16.22	4.00	25.42	9.46	3.95	17.81	2.92	36.46	11.04
13. Verbal Analogies (Gc)	19.51	5.11	24.73	3.25	18.73	20.06	4.76	26.11	2.66	24.46	5.73
14. Disarranged Sentences (Gc)	10.42	4.12	16.84	3.62	24.88	10.11	3.88	17.38	3.52	29.47	4.59
15. Proverbs Interpretation	6.71	2.36	9.44	2.06	18.53	6.93	2.37	9.89	1.98	20.41	1.88
16. Arithmetic	6.85	3.09	11.76	4.34	19.83	7.20	4.43	13.62	5.42	19.55	−0.27
17. Perceptual Reason. (Gf)	23.84	5.28	33.84	4.43	30.90	22.54	6.62	35.05	4.00	35.34	4.44
18. Pictorial Unbalanced Structure	41.36	9.64	49.94	9.28	13.60	38.18	8.16	53.24	6.08	31.73	18.12
19. Combined Solutions	39.99	10.1	45.73	8.95	9.04	37.47	9.44	49.17	7.41	20.83	11.79
20. Multiple Solutions (Gf)	44.54	6.55	50.68	4.81	16.21	42.55	7.15	51.91	3.70	25.88	9.67
21. Pictorial Poly-profile (Gf)	38.68	6.17	45.44	5.83	16.90	38.03	5.99	48.28	4.46	29.43	12.53
22. Dominoes D-48 (Gf)	26.12	4.99	32.82	4.96	20.20	28.09	4.41	34.62	4.65	21.72	1.52
23. Figure Classification (Gf)	6.62	2.39	9.89	2.20	21.37	6.40	2.41	10.89	2.14	29.60	8.23
24. Projection in Water (Gf)	7.22	1.62	8.12	1.23	9.47	7.00	1.69	8.43	0.92	16.44	6.96
25. Figure Series (Gf)	9.95	2.68	12.31	2.20	14.51	9.42	3.29	13.38	2.32	21.18	6.67
26. Matrices I (Gf)	8.25	1.46	9.34	0.94	13.63	7.85	1.54	9.56	0.74	22.50	8.88
27. Matrices II (Gf)	9.23	1.52	9.99	1.15	8.54	8.24	1.82	10.50	0.79	25.98	17.44
28. Matrices III (Gf)	5.46	2.72	7.51	2.07	12.84	5.46	2.64	8.90	1.66	24.00	11.16
**AVERAGE**	**18.48**	**4.18**	**23.19**	**3.79**	**17.70**	**17.67**	**4.37**	**24.67**	**3.19**	**27.78**	**10.08**

Notes: Gc and Gf refer to crystallized intelligence and fluid intelligence, respectively, as defined in Stankov and Chen (1988a, 1988b). The last column presents the difference between experimental and control groups in the amount of change between the Initial and 2nd Retest scores. Using values in the first row (Essential Features test), the calculations involved (a) assessment of control groups’ mean change from the Initial to the 2nd Retest: 37.49 − 30.76 = 6.73; (b) calculating the average of SDs from the Initial and the 2nd Retest: (5.45 + 4.21)/2 = 4.83; (c) calculating Cohen’s *d_av_* = 6.73/4.83 = 1.393; (d) converting Cohen’s *d_av_* to IQ units 1.393 × 15 = 20.90. Applying the same approach to the data from the experimental group produced 34.59 IQ units change. The value in the last column is the difference between the changes in experimental and control groups: 34.59 − 20.90 = 13.69.
